# Mental Health Factors That Guide Individuals to Engage in Overconsumption Behavior During the COVID-19 Pandemic: A Cross-Cultural Study Between USA and Ecuador

**DOI:** 10.3389/fpubh.2022.844947

**Published:** 2022-03-22

**Authors:** Velasco Franklin, Lanchimba Cintya, Paz y Miño Mariel, Díaz-Sanchez Juan Pablo

**Affiliations:** ^1^Department of Marketing, Universidad San Francisco de Quito USFQ, Quito, Ecuador; ^2^Departamento de Economía Cuantitativa, Facultad de Ciencias Escuela Politécnica Nacional, Quito, Ecuador; ^3^Institut de Recherche en Gestion et Economie Université de Savoie Mont Blanc (IREGE/IAE Savoie Mont Blanc), Annecy, France; ^4^Head of Psychology Department, Director of Mental Health Clinic, Universidad San Francisco de Quito USFQ, Quito, Ecuador; ^5^Tenured Lecturer IDEA Research Group, Departamento de Economía Cuantitativa Escuela Politécnica Nacional, Quito, Ecuador

**Keywords:** COVID-19, health consciousness, overconsumption, wellbeing, hoarding activity, culture, individualism—collectivism

## Abstract

**Background:**

This study tests a framework that examines the role of several mental health factors (mood, wellbeing, health consciousness, and hoarding) on individuals' overconsumption behavior under the novel coronavirus context. This examination is relevant to public health literature because it increases our knowledge on how the context of COVID-19 pandemic affects people's mental health and provides answers to why individuals engage in overconsumption behavior. Additionally, this research also follows a cross-cultural perspective aiming to understand how individuals from different cultural orientations cope with the psychological effects of the COVID-19 pandemic.

**Methods:**

This is a cross-sectional study that compares samples from two countries: Ecuador (*n* = 334) and USA (*n* = 321). Data was collected via an online survey. The timing of data collection was set during the mandatory lockdowns and social distance measures taken by both countries to fight against the COVID-19 virus breakout. Partial least squares structural equation modeling was used to test the theorized framework. Multi-group analysis was used to explore cultural orientation differences among the relationships included in the model.

**Results:**

The results indicate that individuals' mood state has a positive relationship with health consciousness, as people try to regulate their health concerns by maintaining positive perceptions of their subjective wellbeing. Further, the increased concern individuals express in their health is responsible for them to engage in overconsumption behavior. Cultural orientation (individualism vs. collectivism) moderates the relationship between mood and health consciousness. No moderation effect was found for the relationship between health consciousness and overconsumption.

**Conclusions:**

The COVID-19 pandemic has generated negative effects in individuals' mental health. Findings from this study suggest that maintaining a positive mood is important for individuals at the time of mandatory lockdowns, and this effort is related to a greater concern and awareness of their health. Further, health consciousness is responsible to stimulate overconsumption behavior. This chain of effects can be explained by individuals' interest in their wellbeing. Culture plays a role in these effects. People from individualistic countries (USA) compared to people from collectivistic countries (Ecuador) demonstrate greater motivation in maintaining their positive mood by showing greater health consciousness.

## Introduction

The Coronavirus Disease 19 (COVID-19) pandemic has had a tremendous impact on people's lives, especially affecting the mental health of individuals (1). High levels of threat have governed people's sentiments in the marketplace (2). People are experiencing a “collective trauma” in the form of mental health issues such as depression, anxiety, and stress (3–5), and are reflecting on their health habits and overall wellbeing (6). These conditions have caused an unprecedented disruption in the public health, forcing retailers, and households to adapt quickly to the new context (2, 7).

One particular behavior that people have unveiled since the emergence of COVID-19 is overconsumption, such as stockpiling food, medicines, cleaning products, and other essential items (8). Due to overconsumption, the marketplace (e.g., retailers and healthcare providers) have suffered from a lack of supply of goods during the COVID-19 pandemic. Previous literature has pointed out the role of the marketplace in helping consumers to cope with high-threat situations and mental health factors (9). In this study we interpret overconsumption as a tactic used by people to help alleviate the impact of stressor factors associated with the forced lockdowns and social distancing measures related to COVID-19. Moreover, we follow a cross-cultural perspective to identify if differences in cultural orientation at the country level (10) manifest in individuals' mental states, self-regulatory tactics, and coping strategies. These differences can be explained by previous literature describing how culture affects individuals' judgments based on construals of the self and others (11). This cross-cultural perspective is important as it not only considers differences in societies cultural values, but also how countries adopted different measures to prevent and control COVID-19 virus breakout. Drawing from these arguments, this study explores several mental states acting as drivers of people's overconsumption behaviors. Overall, these mental states are predicted to have an influence on individuals' ability to emotionally self-regulate.

More specifically, this study aims to investigate the antecedents of overconsumption behavior by exploring the chain of effects of several mental health factors (e.g., mood, subjective wellbeing, health consciousness, and hoarding tendency). We use a cross-cultural approach to examine the relationships among these factors, considering that people react differently when coping with the COVID-19 outbreak and that mental health characteristics differ from one society to another. We strategically chose two markets to compare the effects, the USA and Ecuador, as they represent the extremes of individualistic and collectivistic cultural orientations (10). Data collection occurred during the mandatory lockdowns and social distancing measures, in April and May 2020, for both countries. The impact of COVID-19 in these countries is alarming, as both are included on top of the rankings of COVID-19 cases and deaths per capita according the World Health Organization (12).

### Mood Impact on Health Consciousness

Mood states represent an important set of affective factors that guide people's behavior in different situations (13). A particularly relevant characteristic of mood is that it is easily influenced by situational factors (14). Individuals subjectively perceive their mood as having a positive (cheerful, peaceful, optimistic) or a negative (anxious, sad, depressed) feeling state, and this state often contrasts with other mental states (13). In this study we examine the link between mood and health consciousness. Health consciousness is defined as the degree to which health concerns are integrated into a person's daily activities (15). Because individuals' mood often guides them to be more attentive to information congruent their feeling state (13), we argue that a positive mood is strategically sustained in people by an increased concern for their health.

For this argument we follow the insights reported by extensive psychology literature that associate mood in generating bias evaluations with mood-congruent directions (16–18). For instance, an individual who has a positive mood would very likely want to protect that positive mental state by selectively making positive judgments about their health situation. Also, we can expect individuals to become more attentive to the beneficial or detrimental aspects of their wellbeing when mood regulation is a priority to them (19). Thus, we are predicting that mood has a positive relationship with health consciousness.

**H**_**1**_ Positive mood is positively associated with health consciousness.

### Influence of Health Consciousness on Overconsumption

When people are asked about their health, they use a reference point for comparison (19). For example, individuals might compare their current health with a pre COVID-19 health state. Depending on the situation, this mental process may result in either relief or concern feelings. Mujcic and Frijters (18) interpreted health consciousness with an economic utility function—that is, perceiving gains (e.g., “I feel healthy”) or losses (e.g., “I feel concern about my health”). The authors further discuss that people often experience a “shock” when feeling no control over their health situation. Therefore, it is expected that at higher levels of health concern, people will start having a notion of deterioration of their subjective wellbeing. Interestingly, this cognitive process generates in individuals a strong motivation to strive toward recovering the health spirit or at least to try to sustain it through their *resource expectations*. This process reveals that individuals try to regulate their health concerns by applying approach or avoidance coping tactics. We propose that one of those tactics is engaging in overconsumption, which refers to purchasing and consuming goods in an excessive manner (20). Overconsumption involves a mental calculation practice that is deliberative in people and responds to marketplace practices and situations (20), such as accumulating large quantities of essential goods during mandatory lockdowns to “feel better” about the self. These high levels of health concern correspond to the COVID-19 threat and individuals' lack of power to freely purchase goods when they are ordered to stay at home and maintain social distancing. By strategically focusing on overconsumption, we indirectly point to the marketplace as being a relevant instrument for people to engage their motivation to sustain their mental wellbeing (21).

Three studies provide insights on how overconsumption serves as a tactic for individuals to cope with negative mood. A recent study shows that individuals are willing to purchase extra bottles of a sport drink when experiencing negative feelings (22). Another study shows how individuals are willing to engage in purchasing more food products that help improve one's state of health when their health consciousness motivates them to improve their wellbeing (9). Also, there is evidence that health concern could be interpreted as a personal value—an enduring belief about what is fundamentally important (23). As such, health consciousness could be conceptualized as a possession or a unique resource that induces overconsumption behaviors in an individual experiencing chronically high levels of concern.

In sum, we predict that health consciousness has a positive relationship with overconsumption.

**H**_**2**_ Health consciousness is positively associated with overconsumption.

### The Mediating Effect of Subjective Wellbeing

Subjective wellbeing refers to an individual's mental state, characterized by the articulation of positive or negative thoughts about the self and by expressing an overall assessment of the degree of satisfaction about different aspects of the individual's life (24, 25). Subjective wellbeing also has been used to describe an individual whose stability, coping skills, happiness, confidence, and sense of being grounded (26) contribute to their perseverance in the face of challenges, providing a combination of “feeling good and functioning effectively” (27). This happens because optimal mental health is conceived as a complete state of wellbeing when emotions are under control (28).

What is interesting about subjective wellbeing is that it acts as an important motivational resource for individuals to regulate their emotions and mental health outcomes (e.g., stress, depression, and health concerns) (26–29). We argue that subjective wellbeing is the psychological mechanism that drives individuals' efforts to try to sustain their positive mood when experiencing increased health consciousness due to the threat of COVID-19.

Behavioral researchers use the term *positive psychological capital* to define this facet of people's pursuit of their wellbeing (30). Subjective wellbeing is a mental state characterized by putting in the necessary effort in challenging times (self-efficacy): showing confidence, resilience, and optimism and having hope when adversity is present in individuals' lives (27). When people perceive having control of their mental health, they are more inclined to use adaptive strategies to cope with everyday emotions (31). These tactics result in individuals' exhibition of approach or avoidance attitudes or behaviors to cope with certain mental health issues (31). For example, people's ability, effort, and focus could be staying alert to their health status in order to suppress negative wellbeing outcomes (i.e., psychological distress and anxiety related to the forced lockdowns and social distancing measures provoked by COVID-19).

Even though the human brain is wired to use past experiences and coping skills as mechanisms to increase the odds of adapting to threat situations or obtaining desired outcomes (32), the COVID-19 pandemic has presented unprecedented mental health issues (e.g., isolation, lockdown, social distancing, and trauma) and unending uncertainty. In fact, the great majority of individuals have not been able to maintain a lifestyle that contributes to their wellbeing (1, 3, 33). As a consequence, the anxiety resulting from people's efforts to sustain their wellbeing has instead generated *maladaptive responses* and *coping behaviors* that contradict their emotional wellbeing. Therefore, we predict that one of these maladaptive responses or coping behaviors is expressed in the form of excessive health concerns.

**H**_**3**_ Subjective wellbeing mediates the relationship between mood and health consciousness.

### The Mediating Effect of Hoarding Tendency

When increased feelings of uncertainty and threat exist around public health, it is common to find in individuals a rise in hoarding tendency (34). Hoarding is a compulsive behavior to purposely engage in repetitive purchases to accumulate goods in an excessive manner when negative events or feelings are salient in people's daily lives (35). Moreover, hoarding is considered a type of mental disorder, as it is an expression of obsessive and compulsive levels of anxiety (36). When people use hoarding to cope with uncertainty (i.e., high levels of concern for one's health), their behavior in the marketplace is demarcated as an automatic reaction characterized by rapid decision-making processes, by displaying a decreased sensitivity approach, and by an urgent need for immediate possession of goods (34).

In high-threat situations, like the COVID-19 pandemic, the levels of anxiety and stress in public health exacerbate the fear of not accumulating essential products, as people have scarce opportunities to shop during lockdowns (36). As a result, individuals regulate their mental states (e.g., health concerns) by engaging in overconsumption.

**H**_**4**_ Hoarding mediates the relationship between health consciousness and overconsumption.

### The Moderating Effect of Individualism (vs. Collectivism)

Culture is known to influence how people perceive, express, and experience the link between emotions and mental distress (37). Furthermore, those beliefs seem to affect how individuals react to their level of concern for their health and mental distress (38). As mentioned by Kowal et al. (39) the role of culture has been widely studied for decades and debates are still going on in terms of how cultural factors may act as a buffer to the environmental stressors or, on the contrary, exacerbate stress levels (39). Since in individualistic societies people care most about the self (9, 37), it is expected that individualists would demonstrate a deeper level of motivation to sustain their positive mood and health. On the other hand, in collectivistic societies people care more about others (9), so it could be expected that their inner motivation to sustain a positive mood and health will be weaker compared to individualists. However, we must not forget that acting only on one needs pleasure, leads to more stress for individuals in quarantine times. We propose that an individualistic (vs. collectivistic) cultural orientation exacerbates or attenuates the effects of the relationships between the constructs included in the model. In particular, we focus on the moderating effect in the following two links: (a) the relationship between mood and health consciousness, and (b) the relationship between health consciousness and overconsumption.

Culture serves as natural guidance for people on how to deal with their value-identification processes (40, 41). People in individualistic societies are individual-centric and demonstrate a resilient orientation toward autonomy and self-efficacy (9, 42). In individualistic societies people prioritize their own interests and goals over those of the group (40). Individuals in individualistic societies by default focus on personal wellbeing and their material needs compared to individuals from collectivistic societies (37). However, it should be stressed that during the current quarantine, people have been forced to renounce their personal enjoyment (e.g., sports, concerts, shopping, travel, social gatherings) for the sake of group needs (39). In fact, it would be expected that the more individualistic individuals are, the higher the chances they would not adhere to epidemic prevention measures (43). Thus, individualistic societies might maintain mood and health consciousness by prioritizing their needs over the collective health of society, thus generating, as stated by Maaravi et al. (43), higher chances of not adhering to epidemic prevention measures, less vaccination and more death tolls.

On the other hand, people living in collectivistic societies demonstrate being interdependent with their community and assign relevance to social norms when forming their attitudes and engaging in consumption behaviors (9, 42). An important characteristic of collectivistic societies, key to the scope of this study, is that individuals in these societies are willing to make personal sacrifices because in their consumption goals interest is placed in the society's wellbeing (42). Thus, for collectivistic people, others' welfare is as highly relevant as their own welfare, while individualistic people care about the private self.

**H**_**5**_ Individualism (vs. collectivism) moderates the relationship between mood and health consciousness.

Hofstede (44) labeled individualistic societies, with people's strong concern about the self and immediate family, and as having an emphasis on self-fulfillment as a characteristic. Another significant characteristic of individualistic societies is how people in these societies strive for norms like *living up to one's potential* (40, 42). In this sense, individualistic societies are fundamentally transaction oriented (e.g., purchasing goods at their own will guided by their self-interest). Given these characteristics, we propose that people living in individualistic countries will exhibit more overconsumption when their health concerns are dominant in their minds. This happens because individualism implies one's effort to accumulate resources (i.e., food, medicines, vitamins, cleaning products, and so forth) and having them at immediate disposal to deal with one's self-interests (e.g., deal with one's health concerns provoked by COVID-19-related forced lockdowns and social distancing measures). Therefore, as described by Kowal et al. (39) it might be reasonable to think that the emotional cost of this quarantine period would be greater in individualistic cultures (39). In fact, collectivistic (vs. individualistic) cultures put more emphasis on group harmony over personal interests and enjoyment (9).

At the other extreme, people in collectivistic societies think about others before taking action. The social norm of “being obliged” to others, salient in collectivistic societies (38–40), can cause people in these societies not to engage as much in overconsumption when coping with health concerns. Because collectivism is characterized by a communal orientation, with people having a mindset for the common good and a focus on maintaining harmony and avoiding conflicts with others (9, 40), it is reasonable to expect their priority will not be stockpiling goods. Thus, this expression will indirectly evidence a lower likelihood of using the marketplace to cope with their health concerns. Collectivism societies strive for group harmony; thus, less stress is developed in the process of helping others (9).

**H**_**6**_ Individualism (vs. collectivism) moderates the relationship between health consciousness and overconsumption.

## Materials and Methods

### Sample Characteristics and Data Collection

The reference population for this study is American and Ecuadorian customers. According to Hofstede (9), the USA has a highly individualistic cultural orientation, while Ecuador is high in collectivism (44). Institutional Review Board (IRB) approved this study's procedure for using an anonymous, Internet-based, cross-sectional survey. All participants were informed that their participation was voluntary and consent was implied when accepting to answer the questionnaire. Participants were invited through social media channels and email invitations to fill out a survey concerning the impact of COVID-19 on their mental health and consumption habits. Snowball and convenience sampling were used to recruit participants. Data collection took place between April and May 2020, a time when both countries were heavily impacted by the virus outbreak and government implementation of mandatory lockdown restrictions and social distancing measures. Two surveys were designed for the present study: one for American customers and another for Ecuadorian customers. We used the *back-translation technique* (45) to translate the questionnaire into Spanish for data collection in Ecuador. Three waves of social media invitations to participate in the study were sent using the institutional accounts of a private mental health clinic and the universities' accounts. After screening participants and identifying them as those who usually shop for themselves or their families, the final sample was made up of 655 participants, mean age of 34.88 years (SD = 12.28). The sample of American customers includes 321 participants with a mean age of 29.47 years (SD = 10.95), and 46% were female. Meanwhile, the sample of Ecuadorian customers is 334 with a mean age of 39.98 years (SD = 11.27), and 53% were female. Apart from age and gender, other demographic variables such as number of household members and employment status were collected. Table 1 summarized the sample characteristics.

**Table 1:** Sample demographic characteristics.

**Characteristics**	**No. (and %) of respondents**	
	**Ecuador**	**USA**
**Sex**		
Male	171 (51.20)	118 (36.76)
Female	163 (48.80)	203 (63.24)
**Respondent age**		
Young adults (18–34 y)	92 (27.54)	187 (58.26)
Middle adults (35–49 y)	194 (58.08)	110 (34.27)
Old adults (50–64 y)	31 (9.28)	22 (6.85)
Elders (>65 y)	17 (5.09)	2 (0.62)
**No. Household members**		
Children (0–12 y)	112	78
Teenagers (13–19 y)	135	127
Adults (20–65 y)	270	291
Older adults (>65 y)	25	17
**Employment status**		
Employed	219 (65.57)	205 (63.86)
Unemployed	115 (34.43)	116 (36.14)

### Measures

Our literature review of the constructs included in the model provides the basis for the design of the questionnaire. Scale adaptations from previous marketing studies on mental health, overconsumption, and psychological factors were used. All items were measured using a seven-point scale. All items and their validity scores are listed in Table 2.

**Table 2:** Study's measures and indicators.

**Composite/Indicators**	**Indicator loading**	**AVE**	**Composite reliability**	**Cronbach's alpha**
**Mood** **(****52****)**		0.873	0.932	0.854
I am in a good mood.	0.930			
I feel happy.	0.939			
At this moment I feel nervous or irritable.				
**Hoarding Tendency** **(****53****)**		0.784	0.916	0.863
Getting rid of stuff is difficult for me.	0.903			
I tend to hold on to my possessions.	0.890			
Unless I have a really good reason to throw something away, I keep it.	0.863			
**Health Consciousness** **(****54****)**		0.584	0.807	0.652
I'm alert to changes in my health.	0.736			
I'm concerned about the health of others.	0.817			
Throughout the day I am aware of what foods are best for my health.	0.735			
**Overconsumption** **(****21****)**		0.605	0.821	0.677
Comparing what is happening now vs. pre-COVID-19 “I buy more than before”				
Medicines and vitamins	0.766			
Cleaning products	0.835			
Groceries	0.728			
**Subjective well-being** **(****23**, **24****)**		0.990	0.997	0.995
Now, I appreciate more the life that I had before.	0.995			
Now, I can do things that I didn't do before.	0.995			
I should totally change my lifestyle as soon as this ends.	0.995			
**Covid**				
Do you have a relative or friend who has been diagnosed with COVID-19? (Yes or No)	1.00			
Do you have any relative or friend who is high risk of COVID-19 contagion? (Yes or No)	1.00			
Do you know someone, close to you, that died from COVID-19? (Yes or No)	1.00			

To address the potential for common-method bias in our study we ran two tests. We used Kock's (46) full collinearity test for common-method bias in Partial Least Squares Structural Equation Modeling (PLS-SEM) models (46). This test resulted in none of our items showing a VIF higher than 3.3, as they ranged between 1.07 and 2.71. Thus, the test results were optimal.

### Data Analysis

In order to test the proposed model and hypotheses we used PLS-SEM to simultaneously assess the measurement and the structural model, and to estimate the differences between the path coefficients of the USA and Ecuador models. PLS-SEM is considered a reliable data analysis technique to study relationships among variables and is considered suitable to test exploratory models (47, 48). Smart Partial Least Squares SmartPLS version 3.3.2 software (49) was used to compute the items' psychometric properties and factor loadings, as well as to estimate model fit statistics, compute path coefficients, and perform multi-group analysis.

## Results

### Measurement Model

First, we evaluated the psychometric properties of the constructs included in the model. Convergent validity was assessed by the average variance extracted (AVE) scores and composite reliability (CR) for all variables. AVE scores were above the 0.5 threshold as Hair et al. (47) recommended (47). Second, we found that the CR scores for all constructs were robust and above 0.8. Third, the constructs demonstrated adequate reliability indices, as Cronbach's alpha for all constructs was above 0.7. To further check the reliability of the constructs, we followed Henseler et al.'s (50) recommendation and confirmed that the heterotrait-monotrait indices were below the maximum value of 0.9 (50). Finally, all outer loadings were significant and the rho_A indicators were higher than 0.7 (51). Table 2 summarizes the constructs' psychometric properties.

Then we performed the analysis for the discriminant validity. All tests were successful. The average shared variance of each construct and its diagonal values, illustrated in bold in Table 3, exceed the shared variance with other constructs (Fornell-Larcker criterion). Table 3 shows the heterotrait-monotrait ratio (HTMT) above the diagonal, the square root of the AVE in the diagonal (bold), and correlations between the constructs under the diagonal.

**Table 3:** Discriminant validity.

**Construct Name**	**O**	**CO**	**HC**	**H**	**MO**	**W**
Overconsumption (O)	**0.778**					
COVID-19 (CO)	0.031	**0.923**				
Health consciousness (HC)	0.202	0.177	**0.764**			
Hoarding (H)	0.145	0.156	0.172	**0.886**		
Mood (MO)	0.164	0.244	0.148	0.028	**0.934**	
Wellbeing (W)	0.091	0.827	0.137	0.279	0.335	**0.995**

### Structural Model

We first followed the steps recommended by Evermann and Tate (55) and Shmueli et al. (56) to assess the goodness-of-fit of the model by evaluating it with the partial least squares predict *PLSpredict* metric (55, 56). Results from this analysis demonstrate the predictive power of our model since all indicator values were above zero. In addition to this indicator, we checked if the standardized root mean square residual coefficient (SRMR) demonstrates that the model has an adequate fit. The SRMR of the model is 0.06, which is below the threshold of 0.08 suggested by Henseler et al. (57). Apart from SRMR, other indicators for model fit demonstrated the robust predictive performance of our model (d_ULS = 0.77; d_G = 0.286; Chi-Square = 187.04; and NFI = 0.88).

**Figure 1:**
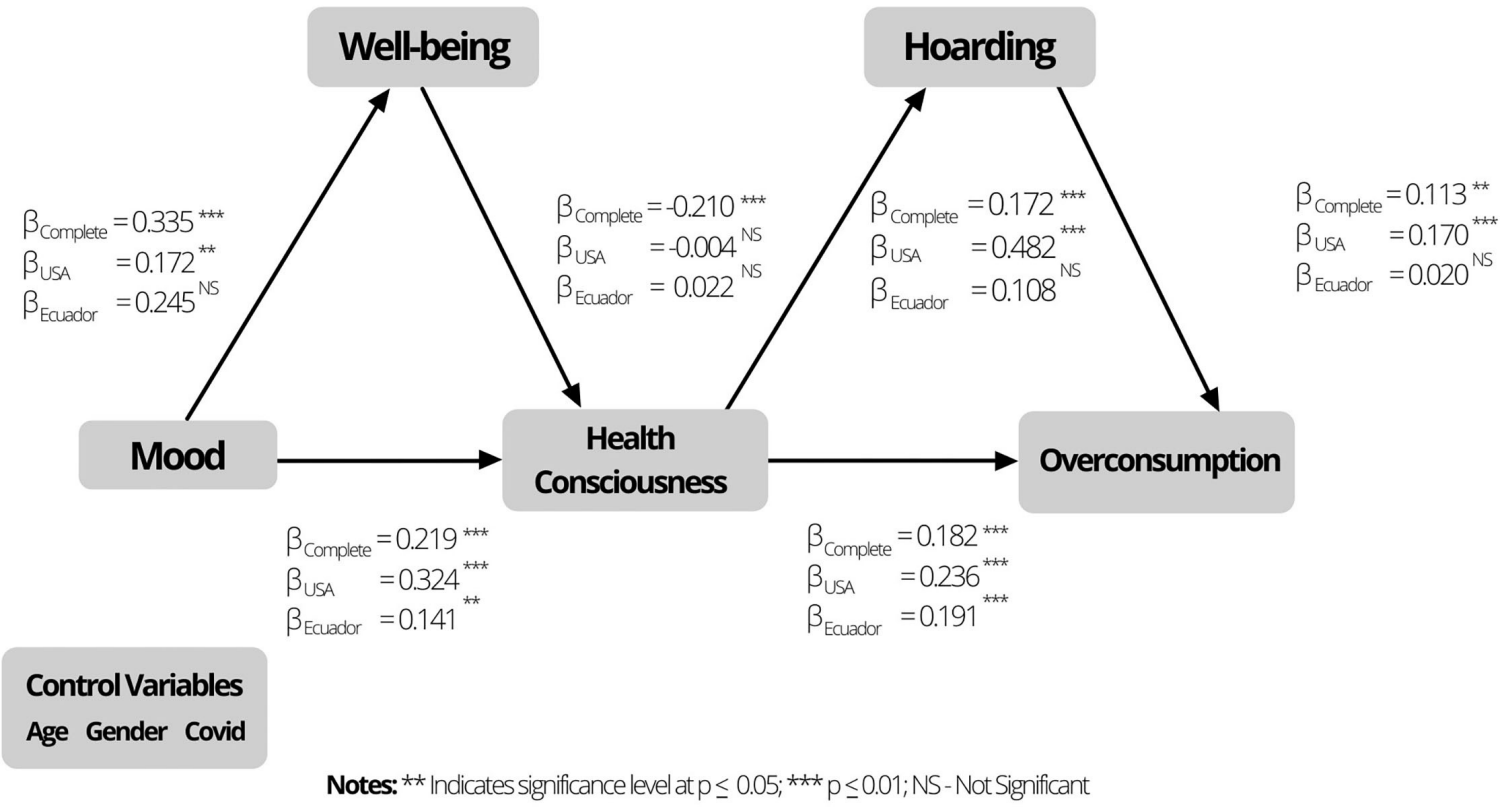
Model's path coefficients.

Second, hypothesis testing was performed by computing the path coefficients among the constructs included in the model. These path coefficients and statistic tests are included in Table 4. H_1_ states that customers' mood influences the level of health consciousness. The path coefficient (β = 0.219, *p* < 0.001) supports our hypothesis. We also found supporting evidence that health consciousness has a positive relationship with overconsumption behavior, as the path coefficient (β = 0.182, *p* < 0.001) is positive and statistically significant. Thus, H_2_ was supported. Table 4 presents the path coefficients for the structural model. Figure 1 illustrates the model's path coefficients.

**Table 4:** Path coefficients.

**Paths**		**Complete model**			**USA**			**Ecuador**	
	**β**	***t*** **-statistic**	***P*** **-value**	**β**	***t*** **-statistic**	***P*** **-value**	**β**	***t*** **-statistic**	***P*** **-value**
**H**_**1**_ Mood → Health consciousness	0.219	4.392	0.000	0.324	4.856	0.000	0.141	2.178	0.030
**H**_**2**_ Health consciousness → Overconsumption	0.182	3.956	0.000	0.236	3.547	0.000	0.191	2.677	0.008
Mood → Wellbeing	0.335	9.774	0.000	0.172	2.442	0.015	0.245	1.414	0.158
Wellbeing → Health consciousness	−0.210	5.052	0.000	−0.004	0.075	0.940	0.022	0.150	0.880
Health consciousness → Hoarding	0.172	4.235	0.000	0.482	9.437	0.000	0.108	1.103	0.271
Hoarding → Overconsumption	0.113	2.566	0.011	0.170	2.849	0.005	0.020	0.232	0.817

Third, we focused on the mediation effects included in the model. Hypothesis 3 (H_3_) predicts that subjective wellbeing mediates the relationship between mood and health consciousness. The indirect effect (β = −0.070, *p* < 0.001) confirms this prediction. This mediation effect was also examined by performing a more elaborate mediation test, using Preacher and Hayes's (58). Model 4 method (58). Consistent with our expectations, we found a statistically significant indirect effect of wellbeing (β = 0.04, SE = 0.01, CI = [0.02–0.06], *p* < 0.001) that provides evidence of a partial mediation of wellbeing. Hypothesis 4 (H_4_) predicts that hoarding mediates the relationship between health consciousness and overconsumption. The indirect effect (β = 0.020, *p* < 0.05) confirms this prediction. Further examination for this mediation effect, using a statistical approach similar to that used for H_3_, resulted in a statistically significant indirect effect of wellbeing (β = 0.03, SE = 0.01, CI = [0.01–0.06], *p* < 0.001) that provides evidence of a partial mediation of hoarding. Table 5 includes the direct and indirect effects statistics of our further analysis of the mediation effects.

**Table 5:** Mediation analysis results.

**Structural relationships**	**Indirect effect**	***t*** **-value**
**H**_**3**_ Mood → Wellbeing → Health consciousness	0.25^***^	2.18
**H**_**4**_ Health consciousness → Hoarding → Overconsumption	0.22^***^	2.54
Mood → Health consciousness → Overconsumption	0.03^*^	2.12
**Mediation effect of Wellbeing (Process Macro, Model 4 indicators)**		
Direct effect mood → Health consciousness without mediator	0.12^***^	4.04
Direct effect mood → Health consciousness with mediator	0.08^**^	2.59
Indirect effect	0.04^***^	
**Mediation effect of Hoarding (Process Macro, Model 4 indicators)**		
Direct effect Health consciousness → Overconsumption without mediator	0.29^***^	5.06
Direct effect Health consciousness → Overconsumption with mediator	0.26^***^	4.52
Indirect effect	0.03^***^	

*** *p ≤ 0.001;*

** *p ≤ 0.01;*

* *p ≤ 0.05*.

Although the focus of the study is on the hypothesized mediating effects, the model implicitly proposes health consciousness as the mediator between mood and overconsumption. The indirect effect (β = 0.030, *p* < 0.05) provides support for this mediating effect.

### Multi-Group Analysis

The multi-group analysis was the statistical technique we used to test the moderating effect of cultural orientation (i.e., individualism vs. collectivism). We followed the steps recommended by Matthews (59) when performing this analysis (59). The first step was to identify and divide the study population into two groups of interest (individualistic and collectivistic cultural orientation) based on participants' nationality: US participants and Ecuadorian participants. The second step was to confirm the existence of invariance between the two groups (50, 59). We applied the measurement invariance of composite models (MICOM) procedure to compare the explained invariance for the USA and Ecuador groups. These initial tests were successful, as the original correlations are greater than or equal to the 5% quantile. Furthermore, full invariance was established, as the mean original difference values and variance original values fall between their corresponding 95% confidence intervals. The third step was to estimate the differences between path coefficients for both subsamples using permutation (59). When these differences are statistically significant, an individualistic (vs. collectivistic) cultural orientation is found to have a moderating effect. The results indicate that cultural orientation (individualism vs. collectivism), determined by country (USA vs. Ecuador), moderates three relationships, as they are significantly different when comparing the two groups. Table 6 shows the results of the multi-group analysis.

**Table 6:** Multigroup analysis.

**Paths**	**β_USA_ (1)**	**β_Ecuador_ (2)**	**B Permutation Difference (1,2)**	**2.50%**	**97.50%**	**Permutation *P*-values**
**H**_**5**_Mood → Health consciousness	0.324	0.141	0.183	−0.204	0.207	0.100
**H**_**6**_Health consciousness → Overconsumption	0.236	0.191	0.045	−0.176	0.172	0.818
Mood → Wellbeing	0.172	0.245	−0.073	−0.137	0.133	0.295
Wellbeing → Health consciousness	−0.004	0.022	−0.026	−0.171	0.172	0.182
Health consciousness → Hoarding	0.482	0.108	0.374	−0.157	0.163	0.001
Hoarding → Overconsumption	0.170	0.020	0.150	−0.163	0.168	0.033

Following Matthews's (59) suggestion to use the permutation *p*-value as a test to identify significant differences between groups, it is evident that there is a marginally significant difference between the two groups in the impact of mood on health consciousness. In individualistic societies this effect is stronger compared to collectivistic societies (β_USA_ = 0.324, β_Ecuador_ = 0.141, *Permutation p*-value = 0.100). This finding provides partial evidence to support H_5_.

There was no statistically significant difference between the USA and Ecuador groups in the impact of hoarding on overconsumption. The effect of the relationship between hoarding and overconsumption does not differs between individualistic and collectivistic societies (β_USA_ = 0.236, β_Ecuador_ = 0.191, *Permutation p*-value = 0.818). Therefore, H_6_ was not supported.

Interestingly, we found a significant difference between the two groups in the impact of health consciousness on hoarding (β_USA_ = 0.482, β_Ecuador_ = 0.108, *Permutation p*-value = 0.001). Additionally, we found a significant difference between the two groups in the impact of hoarding on overconsumption (β_USA_ = 0.170, β_Ecuador_ = 0.020, *Permutation p*-value = 0.033).

## Discussion

COVID-19 has affected countless people all around the world (1, 5). Recent statistics count more than 95 million cases and more than 2 million deaths globally (10). The consequences of the COVID-19 crisis affect both individuals and the economy. In fact, the increase in positive cases forced governments to continue to take drastic measures such as lockdowns to avoid the spread of the virus in their respective countries. Although these measures have had a positive impact on containing the virus, the effects on the mental health in people are visible (1, 5). In this study we examine several mental health factors and their influence on overconsumption behavior. We explore the chain of effects among mood states, health consciousness, subjective wellbeing, and hoarding, and their impact on overconsumption behavior, using a cross-cultural approach.

The aim of this study was to test a theoretically model focused on understanding overconsumption behavior in times of COVID-19. Based on a unique dataset with data collected in the USA and Ecuador, we find that a positive mood is positively associated with health consciousness. This result is congruent with previous studies that report how individuals' behaviors are guided by their mood state (13). Thus, in times of COVID-19 individuals demonstrating positive mood are motivated to maintain those positive feelings by staying alert of their health situation. Our results also provide evidence that health consciousness is positively associated with overconsumption. Therefore, our results indicate that individuals use overconsumption behavior as tactic to cope with the stress (e.g., avoid changes in their mood and stay healthy) associated to the pandemic. This result is in line with previous studies that suggest how individuals use their consumption behavior (e.g., panic buying) to maintain their wellbeing (9, 21).

We provide partial evidence for the mechanisms included in the model. First, our results indicate partial evidence for subjective wellbeing as the mechanism that drives individuals to sustain their positive mood though higher health consciousness. Because subjective wellbeing is about maintaining positive thoughts about the self (24, 25), individuals are in need to maintain stability and control over themselves (30). Thus, this motivation seems to be responsible for the positive relationship between one's mood and health consciousness. In addition, we provide partial evidence for hoarding as the psychological determinant that drives people to engage in overconsumption when they try to cope with health concerns. In contexts with increased feelings of uncertainty hoarding tendency rises as a way to accumulate resources (34). Our results suggest that hoarding acts as the coping strategy for individuals to engage in overconsumption to sustain their health. This is consistent with previous research that suggests that hoarding is a key psychological mechanism that drive people's behavior (36).

Regarding the role of culture, our data provide evidence that culture is a moderating factor of several relationships among the mental health factors included in the model. Our focus was on two relationships. First, we found that in individualistic societies, like the USA, people have a strong desire to protect their positive mood through elevating their concern for their health. In collectivistic societies, like Ecuador, this effect is significantly weaker. Interestingly, we found no support for the moderating effect of individualism (vs. collectivism) on the relationship between health consciousness and overconsumption. We might explain this finding as a demonstration of how COVID-19 has negatively impacted the mental health of people (1, 3), no matter where they live or what culture they belong to.

This study opens up a new conversation, as it raises a question. While it is true that a positive mood is generated by responding to one's own (individualistic) demands (40) and generating over-consumption, there is an important degree of stress that this attitude could generate (39, 43). That is to say, we are talking about people who belong to an individualistic society as a self-regulated person-, but at what price? Or from whom?

On the other hand, collective societies that strive for the common good (9) have a lower level of health consciousness. This notion could be explained by how collectivistic societies adhere more to first think about others instead of the self (9). Thus, the levels of virus contagions are reduced as individuals don't want to feel guilty about spreading the virus to others. In collectivistic societies where, communal goals are a priority over achieved of individual interest, we can expect less levels of stress about individual health consciousness. Thus, it is valid to question what is better? What is worse? Do governments and societal institutions should position their public messages to counter COVID-19 virus breakout using claims that emphasize in care for others or care for the self. Thus, this study opens this and other research questions that future research might want to address.

### Theoretical Contribution

This study makes numerous contributions to public health literature. First, it builds on the extant literature linking mental health and compulsive behaviors (20–22, 39, 43). Regarding people's efforts to regulate their feelings and emotions in congruence with their positive mood states, our findings suggest that subjective wellbeing and hoarding are relevant factors on which individuals rely to cope with uncertainty and threat contexts like the ones produced by the COVID-19 crisis. We also indirectly find evidence that the marketplace constitutes an important tool for people when dealing with high levels of health concern and mental distress. When individuals engage in overconsumption they rely on the marketplace to cope with their anxiety and accumulate essential products to alleviate health concerns. This is in line with previous research that highlights the role of the marketplace in support of individuals mental health (20). Thus, this study links the mood-maintenance and mood-congruence consumer psychology literature with actual empirical evidence for compulsive behaviors produced by negative public health contexts.

Second, our study also adds to the developing stream of research on health consciousness, subjective wellbeing, hoarding, and overconsumption. It complements the research body of cross-cultural effects in mood-regulation studies (60–62). Our results suggest that culture moderates the effects of the relationships between mood and health consciousness, between health consciousness and hoarding, and between hoarding and overconsumption.

Third, our study reports differences on how culture affects individuals' judgments based on construals of the self and others. This cross-cultural perspective we followed in this study not only considers differences in societies cultural values, but also how countries adopted different measures to prevent and control COVID-19 virus breakout. Therefore, we provide empirical results that observe how macro-level decision making from governments and local authorities related to control or maintain public health have an influence on individuals' mental health and consumption behaviors (e.g., panic buying and overconsumption). Recent COVID-19 research is reporting similar effects (63–66) that support our findings.

### Implications for Public Health

Our results also have important practical implications. First, we show that in the context of COVID-19 people's mental state impacts overconsumption behavior, even in different countries like the USA and Ecuador. Subjective wellbeing requires redoubled attention from public health institutions since it drives individuals' efforts to sustain a positive mood by increasing their health consciousness.

Our findings also indicate that individuals' hoarding tendencies are responsible for the compulsive behaviors that cause supply shortages. Very often, public health officials do not have control over the continuous supply of products. When individuals unnecessarily accumulate essential goods, they contribute to exacerbate the anxiety imposed by COVID-19 by increasing the levels of mental distress for households who find empty shelves. Further discussion of the relevance of the supply chain and operations management for the marketplace is crucial. The novel coronavirus has created supply shortfalls for many products, and procurement departments from public health institutions must use new techniques to quickly find new suppliers at the lowest operational costs.

People's stockpiling behavior exhibiting around the world could be expected to generate gains for consumer goods manufacturers and retailers. However, for the general public health, overconsumption causes higher levels of anxiety and stress as people strive for maintaining their mental wellbeing. Certainly, public health campaigns are recommended to use communication themes and messages highlighting the common good and demonstrating that overconsumption leads to increased anxiety.

### Limitations and Future Research

This study is not free of limitations. First, our results might be exclusively related to the specific case of the US and Ecuadorian markets. It would be interesting to address our same research questions in different empirical contexts—that is, with different nationalities or cultural values. Second, we dealt with a cross-sectional dataset. Perhaps a longer perspective (i.e., repeating the survey once the pandemic is controlled) would provide complementary results. Third, we assume that both countries under consideration (US and Ecuador) adopted similar measures and length of lockdowns which might not be exactly the case although the data collection process took place during lockdowns. Fourth, our study might suffer from typical limitations of cross-cultural research that uses country-level as unity of analysis to identify differences in cultural orientations. Future research may consider to replicate our findings using an individual-level unit of analysis.

## Data Availability Statement

The raw data supporting the conclusions of this article will be made available by the authors, without undue reservation.

## Ethics Statement

The studies involving human participants were reviewed and approved by Comite de Bioetica Universidad San Francisco de Quito. Written informed consent was not provided because this was an online survey, informed consent was approved electronically.

## Author Contributions

Data collection duties was equality distributed among all authors. VF and LC performed the data analysis. All authors contribute equally to plan the scope of the research and develop the research questions, to the writeup of the manuscript, and gave final approval of the version to be submitted to the journal.

## Funding

This study received funding from Universidad San Francisco de Quito.

## Conflict of Interest

The authors declare that the research was conducted in the absence of any commercial or financial relationships that could be construed as a potential conflict of interest.

## Publisher's Note

All claims expressed in this article are solely those of the authors and do not necessarily represent those of their affiliated organizations, or those of the publisher, the editors and the reviewers. Any product that may be evaluated in this article, or claim that may be made by its manufacturer, is not guaranteed or endorsed by the publisher.

## References

[1] Pereira-Sanchez V Adiukwu F El Hayek S Bytyçi DG Gonzalez-Diaz JM Kundadak GK. COVID-19 effect on mental health: patients and workforce. Lancet Psychiatry. (2020) 7:e29–30. 10.1016/S2215-0366(20)30153-X 32445691 PMC7239628

[2] Pantano E Pizzi G Scarpi D Dennis C. Competing during a pandemic? Retailers' ups and downs during the COVID-19 outbreak. J Bus Res. (2020) 116:209–13. 10.1016/j.jbusres.2020.05.036 32501307 PMC7241368

[3] Diaz-Sanchez JP Lanchimba C Pazymino M Velasco Vizcaino F. La Cuarentena de los Ecuatorianos. (2020). Available online at: https://online.pubhtml5.com/ookt/fbez/ (accessed September 4, 2020).

[4] Kahil K Cheaito MA El Hayek R Nofal M El Halabi S Kudva KG. Suicide during COVID-19 and other major international respiratory outbreaks: a systematic review. Asian J Psychiatr. (2021) 56:102509. 10.1016/j.ajp.2020.102509 33418284 PMC7764387

[5] Ransing R Adiukwu F Pereira-Sanchez V Ramalho R Orsolini L Teixeira ALS. Mental health interventions during the COVID-19 pandemic: a conceptual framework by early career psychiatrists. Asian J Psychiatr. (2020) 51:102085. 10.1016/j.ajp.2020.102085 32413616 PMC7195073

[6] Awan HA Aamir A Diwan MN Ullah I Pereira-Sanchez V Ramalho R. Internet and pornography use during the COVID-19 pandemic: presumed impact and what can be done. Front Psychiatry. (2021) 12:220. 10.3389/fpsyt.2021.623508 33796031 PMC8007884

[7] Sheth J. Business of business is more than business: Managing during the Covid crisis. Ind Mark Mang. (2020) 88:261–4. 10.1016/j.indmarman.2020.05.028

[8] Hall MC Prayag G Fieger P Dyason D. Beyond panic buying: consumption displacement and COVID-19. J. Serv. Manage. (2020) 32:113–28. 10.1108/JOSM-05-2020-0151

[9] Hofstede G. Culture's Consequences: Comparing Values, Behaviors, Institutions and Organizations Across Nations. Thousand Oaks, CA: Sage publications (2001).

[10] WHO. WHO Coronavirus Disease (COVID-19) Dashboard. (2021). Available online at: https://covid19.who.int/ (accessed January 7, 2021)

[11] Gardner MP. Mood states and consumer behavior: a critical review. J Consum Res. (1985) 12:281. 10.1086/208516

[12] Isen AM Shalker TE. The effect of feeling state on evaluation of positive, neutral, and negative stimuli: when you ‘Accentuate the Positive,' do you ‘Eliminate the Negative'? Soc Psychol Q. (1982) 45:58. 10.2307/3033676

[13] Akhondan H Johnson-Carroll K Rabolt N. Health consciousness and organic food consumption. J Family Consum Sci. (2015) 107:27–32.

[14] Gendolla GHE. On the impact of mood on behavior: an integrative theory and a review. Rev General Psychol. (2000) 4:378–408. 10.1037/1089-2680.4.4.378

[15] Schwarz N Clore GL. Mood as information: 20 years later. Psychol Inq. (2003) 14:296–303. 10.1207/S15327965PLI1403&4_20

[16] Wong MY. Towards a theory of mood function. Philos Psychol. (2016) 29:179–97. 10.1080/09515089.2015.1024830

[17] Tice DM Bratslavsky E Baumeister RF. Emotional distress regulation takes precedence over impulse control: if you feel bad, do it!. J Pers Soc Psychol. (2001) 80:53–67. 10.1037/0022-3514.80.1.53 11195891

[18] Mujcic R Frijters P. Conspicuous consumption, conspicuous health, optimal taxation. J Econ Behav Organ. (2015) 111:59–70. 10.1016/j.jebo.2014.12.017

[19] Fisk G. Criteria for a theory of responsible consumption. J Mark. (1973) 37:24–31. 10.1177/002224297303700206

[20] Machin JE Adkins NR Crosby E Farrell JR Mirabito AM. The marketplace, mental well-being, and me: exploring self-efficacy, self-esteem, and self-compassion in consumer coping. J Bus Res. (2019) 100:410–20. 10.1016/j.jbusres.2018.12.028

[21] Velasco F Jorda R. Portrait of boredom among athletes and its implications in sports management: a multi-method approach. Front Psychol. (2020) 11:831. 10.3389/fpsyg.2020.00831 32528344 PMC7264414

[22] Ahuvia AC Wong NY. Personality and values based materialism: their relationship and origins. J Consum Psychol. (2002) 12:389–402. 10.1016/S1057-7408(16)30089-4

[23] Diener E Suh EM Lucas RE Smith HL. Subjective weil-being: three decades of progress. Psychol Bull. (1999) 125:276–302. 10.1037/0033-2909.125.2.276

[24] Silvera DH Lavack AM Kropp F. Impulse buying: the role of affect, social influence, subjective wellbeing. J Consum Market. (2008) 25:23–33. 10.1108/07363760810845381

[25] Soares JM Sampaio A Ferreira LM Santos NC Marques F Palha JA. Stress-induced changes in human decision-making are reversible. Transl Psychiatry. (2012) 2:131. 10.1038/tp.2012.59 22760555 PMC3410630

[26] Huppert FA. Psychological well-being: evidence regarding its causes and consequences. Appl Psychol. (2009) 1:137–64. 10.1111/j.1758-0854.2009.01008.x

[27] King RB de la Rosa ED. Are your emotions under your control or not? Implicit theories of emotion predict well-being via cognitive reappraisal. Pers Individ Dif. (2019) 138:177–82. 10.1016/j.paid.2018.09.040

[28] Uwakwe R Otakpor A. Public mental health – using the Mental Health Gap Action Program to put all hands to the pumps. Front Public Health. (2014) 2:33. 10.3389/fpubh.2014.00033 24795874 PMC4000990

[29] Luthans F Youssef CM Avolio BJ. Psychological Capital: Developing the Human Competitive Edge. New York, NY: Oxford University Press (2007). 10.1093/acprof:oso/9780195187526.001.0001

[30] de Castella K Goldin P Jazaieri H Ziv M Dweck CS Gross JJ. Beliefs about emotion: links to emotion regulation, well-being, psychological distress. Basic Appl Soc Psych. (2013) 35:497–505. 10.1080/01973533.2013.840632

[31] Grupe DW Nitschke JB. Uncertainty and anticipation in anxiety: an integrated neurobiological and psychological perspective. Nat Rev Neurosci. (2013) 14:488–501. 10.1038/nrn3524 23783199 PMC4276319

[32] Chen RN Liang SW Peng Y Li XG Chen JB Tang SY. Mental health status and change in living rhythms among college students in China during the COVID-19 pandemic: a large-scale survey. J Psychosom Res. (2020) 137:110219. 10.1016/j.jpsychores.2020.110219 32862063 PMC7428432

[33] Shoham A Brenčič MM. Compulsive buying behavior. J Consum Market. (2003) 20:127–38. 10.1108/07363760310464596

[34] Wheaton M Timpano KR LaSalle-Ricci VH Murphy D. Characterizing the hoarding phenotype in individuals with OCD: associations with comorbidity, severity and gender. J Anxiety Disord. (2008) 22:243–52. 10.1016/j.janxdis.2007.01.015 17339096 PMC2577614

[35] Rook DW Gardner MP. In the mood: impulse buying's affective antecedents. Res Consum Behav. (1993) 6:1–28.19229837

[36] Cannito L Stefano A Alessandro B Rocco P Irene C Adolfo D. Temporal discounting of money and face masks during the COVID-19 pandemic: the role of hoarding level. Front Psychol. (2021) 12:642102. 10.3389/fpsyg.2021.642102 34177697 PMC8219851

[37] Markus HR Kitayama S. Culture and the self: implications for cognition, emotion, and motivation. Psychol Rev. (1991) 98:224–53. 10.1037/0033-295X.98.2.224

[38] Bhui K Dinos S. Health beliefs and culture: essential considerations for outcome measurement. Dis Manage Health Outcomes. (2008) 16:411–9. 10.2165/0115677-200816060-0000623479537

[39] Kowal M Coll-Martin T Ikizer G Rasmussen J Eichel K Studzinska A. Who is the most stressed during the COVID-19 pandemic? Data from 26 countries and areas. Appl Psychol Health Well-Being. (2020) 12:946–66. 10.1111/aphw.12234 32996217 PMC7537225

[40] Oyserman D Coon HM Kemmelmeier M. Rethinking individualism and collectivism: evaluation of theoretical assumptions and meta-analyses. Psychol Bull. (2002) 128:3–72. 10.1037/0033-2909.128.1.3 11843547

[41] Oyserman D. Identity-based motivation: implications for action-readiness, procedural-readiness, consumer behavior. J Consum Psychol. (2009) 19:250–60. 10.1016/j.jcps.2009.05.008

[42] Triandis HC Bontempo R Villareal MJ Asai M Lucca N. Individualism and collectivism: cross-cultural perspectives on self-ingroup relationships. J Pers Soc Psychol. (1988) 54:323–38. 10.1037/0022-3514.54.2.323

[43] Maaravi Y Levy A Gur T Confino D Segal S. The tragedy of the commons”: how individualism and collectivism affected the spread of the COVID-19 pandemic. Front Public Health. (2021) 9:627559. 10.3389/fpubh.2021.627559 33643992 PMC7905028

[44] Hofstede G. Country Comparison - Hofstede Insights. Hofstede Insights (2022). Available online at: https://www.hofstede-insights.com/country-comparison/ (accessed January 28, 2022)

[45] Beaton DE Bombardier C Guillemin F Ferraz MB. Guidelines for the process of cross-cultural adaptation of self-report measures. Spine. (2000) 25:3186–91. 10.1097/00007632-200012150-00014 11124735

[46] Kock N. Common method bias in PLS-SEM: a full collinearity assessment approach. Int J e-Collab. (2015) 11:1–10. 10.4018/ijec.2015100101

[47] Hair J Hollingsworth CL Randolph AB Chong AYL. An updated and expanded assessment of PLS-SEM in information systems research. Indust Manage Data Syst. (2017) 117:442–58. 10.1108/IMDS-04-2016-0130

[48] Hair JF Risher JJ Sarstedt M Ringle CM. When to use and how to report the results of PLS-SEM. Europ Busin Rev. (2019) 31:2–24. 10.1108/EBR-11-2018-0203

[49] Ringle C Wende S Becker J. SmartPLS 3. Bönningstedt: SmartPLS (2015). Retrieved from: http://www.smartpls.com

[50] Henseler J Ringle CM Sarstedt M. A new criterion for assessing discriminant validity in variance-based structural equation modeling. J Acad Market Sci. (2015) 43:115–35. 10.1007/s11747-014-0403-8

[51] Dijkstra T Henseler J. Consistent and asymptotically normal PLS estimators for linear structural equations. Comput Stat Data Anal. (2015) 81:10–23. 10.1016/j.csda.2014.07.00824306555

[52] Peterson RA Sauber M. A mood scale for survey research. In: Murphy PE editor. 1983 AMA Educators' Proceedings. Chicago, IL: American Marketing Association (1983). p. 409–14.

[53] Haws KL Naylor RW Coulter RA Bearden WO. Keeping it all without being buried alive: understanding product retention tendency. J Consum Psychol. (2012) 22:224–36. 10.1016/j.jcps.2011.05.003

[54] Gould SJ. Consumer attitudes toward health and health care: a differential perspective. J Consum Affairs. (1988) 22:96–118. 10.1111/j.1745-6606.1988.tb00215.x

[55] Evermann J Tate M. Assessing the predictive performance of structural equation model estimators. J Bus Res. (2016) 69:4565–82. 10.1016/j.jbusres.2016.03.050

[56] Shmueli G Ray S Estrada JMV Chatla SB. The elephant in the room: predictive performance of PLS models. J Bus Res. (2016) 69:4552–64. 10.1016/j.jbusres.2016.03.049

[57] Henseler J Hubona G Ray PA. Using PLS path modeling in new technology research: updated guidelines. Indust Manage Data Syst. (2016) 116:2–20. 10.1108/IMDS-09-2015-0382

[58] Preacher KJ Hayes AF. Asymptotic and resampling strategies for assessing and comparing indirect effects in multiple mediator models. Behav Res Methods. (2008) 40:879–91. 10.3758/BRM.40.3.879 18697684

[59] Matthews L. Applying multi-group analysis in PLS-SEM: a step-by-step process. In: Latan H Noonan R editors. Partial Least Squares Structural Equation Modeling: Basic Concepts, Methodological Issues and Applications. Heidelberg: Springer (2017). p. 219–243.

[60] Maier E Wilken R Schneider H Schneider GK. In the mood to buy? Understanding the interplay of mood regulation and congruence in an international context. Market Lett. (2012) 23:1005–18. 10.1007/s11002-012-9200-7

[61] Luomala HT Kumar R Worm V Singh JD. Cross-cultural differences in mood-regulation: an empirical comparison of individualistic and collectivistic cultures. J Int Consum Market. (2004) 16:39–62. 10.1300/J046v16n04_03

[62] Butler EA Lee TL Gross JJ. Emotion regulation and culture: are the social consequences of emotion suppression culture-specific?. Emotion. (2007) 7:30. 10.1037/1528-3542.7.1.30 17352561

[63] Toh SY Yuan SW Kaur R. Do materialistic consumers buy more during the COVID-19 pandemic?: social consumption motivation of anxious malaysians in the face of existential threat of death. Int J Custom Relat Market Manage. (2022) 13:1–18. 10.4018/IJCRMM.289203

[64] Zhang J Jiang N Turner JJ Pahlevan Sharif S. The impact of scarcity of medical protective products on chinese consumers' impulsive purchasing during the COVID-19 epidemic in China. Sustainability. (2021) 13:9749. 10.3390/su13179749

[65] Islam T Pitafi AH Arya V Wang Y Akhtar N Mubarik S. Panic buying in the COVID-19 pandemic: a multi-country examination. J Retail Consum Serv. (2021) 59:102357. 10.1016/j.jretconser.2020.102357

[66] Naeem M. Do social media platforms develop consumer panic buying during the fear of Covid-19 pandemic. J Retail Consum Serv. (2021) 58:102226. 10.1016/j.jretconser.2020.102226

